# Genome-Wide Association Study Candidate Genes on Mammary System-Related Teat-Shape Conformation Traits in Chinese Holstein Cattle

**DOI:** 10.3390/genes12122020

**Published:** 2021-12-19

**Authors:** Mudasir Nazar, Xubin Lu, Ismail Mohamed Abdalla, Numan Ullah, Yongliang Fan, Zhi Chen, Abdelaziz Adam Idriss Arbab, Yongjiang Mao, Zhangping Yang

**Affiliations:** College of Animal Science and Technology, Yangzhou University, Yangzhou 225009, China; drmudasirnazar457@gmail.com (M.N.); dx120180094@yzu.edu.cn (X.L.); ismailhmk@gmail.com (I.M.A.); numanhashmi@aup.edu.pk (N.U.); dx120170088@yzu.edu.cn (Y.F.); zhichen@yzu.edu.cn (Z.C.); arbabtor@yahoo.com (A.A.I.A.); cattle@yzu.edu.cn (Y.M.)

**Keywords:** mammary system-related traits, single-nucleotide polymorphism, GWAS, FarmCPU model, Chinese Holstein cattle

## Abstract

In the dairy industry, mammary system traits are economically important for dairy animals, and it is important to explain their fundamental genetic architecture in Holstein cattle. Good and stable mammary system-related teat traits are essential for producer profitability in animal fitness and in the safety of dairy production. In this study, we conducted a genome-wide association study on three traits—anterior teat position (ATP), posterior teat position (PTP), and front teat length (FTL)—in which the FarmCPU method was used for association analyses. Phenotypic data were collected from 1000 Chinese Holstein cattle, and the GeneSeek Genomic Profiler Bovine 100K single-nucleotide polymorphisms (SNP) chip was used for cattle genotyping data. After the quality control process, 984 individual cattle and 84,406 SNPs remained for GWAS work analysis. Nine SNPs were detected significantly associated with mammary-system-related teat traits after a Bonferroni correction (*p* < 5.92 × 10^−7^), and genes within a region of 200 kb upstream or downstream of these SNPs were performed bioinformatics analysis. A total of 36 gene ontology (GO) terms and 3 Kyoto Encyclopedia of Genes and Genomes (KEGG) pathways were significantly enriched (*p* < 0.05), and these terms and pathways are mainly related to metabolic processes, immune response, and cellular and amino acid catabolic processes. Eleven genes including *MMS22L*, *E2F8*, *CSRP3*, *CDH11*, *PEX26*, *HAL*, *TAMM41*, *HIVEP3*, *SBF2*, *MYO16* and *STXBP6* were selected as candidate genes that might play roles in the teat traits of cows. These results identify SNPs and candidate genes that give helpful biological information for the genetic architecture of these teat traits, thus contributing to the dairy production, health, and genetic selection of Chinese Holstein cattle.

## 1. Introduction

Mammary system traits play an important economic role in cattle [1]. In dairy cattle, mammary infections are a major problem. In response to harmful effects on animal fitness, udder tissues are the first natural protection against pathogens in the teat canal. Several environmental factors can affect teat canal morphology, and a previous study confirmed the existence of a genetic component [2].

Generally, mammary system-related teat shape confirmation trait variation causes mastitis, reducing milk production and affecting milk flow and quality on dairy cattle farms. Long and thick teats have been identified as a possible risk factor for the development of mastitis. Milk somatic cell count and mastitis resistance were found to have a relationship with udder and teat conformation traits [3,4]. Teat anatomical and functional parameters are thought to have a significant impact on milk flow performance [5]. Animal health identified mastitis as one of Ireland’s most economically significant diseases [6]. Bovine mastitis is a disease that affects a large number of dairy cattle around the world, and it has a significant economic impact due to losses at various stages of the dairy production chain [7,8]. However, genetic selection has been introduced as a possible solution to improve future profitability due to confirmation traits in Chinese dairy cattle production [6,9]. The selection of the teats’ confirmation trait decreased the count of somatic cells and the occurrence of clinical mastitis in dairy animals [10]. Teat traits are linked to the occurrence of clinical mastitis in lactating animals [9]. The heritability of teat diameter range was found to be 0.23, 0.27, and 0.35 in three lactations of US Holstein cows [10,11]. The heritability of teat end shape was estimated at 0.53, 0.44, and 0.56 for the first, second, and third lactation periods in US Holstein cows [12]. Teat conformation traits influence mammary system health and have been used as an indicator trait in a selection index to enhance mastitis resistance selection [7,13]. Due to less heritability and an absence of data, selection for teat and udder health traits in dairy cattle is significantly more difficult than selecting for productivity traits, while recent quantitative trait loci (QTL) mapping studies found QTL to be linked with mammary system-related traits [8,12,13].

Genome-Wide Association Study (GWAS) has confirmed that the single-nucleotide polymorphisms (SNPs) are used as genetic markers to identify the complete genome for target genes that are associated with phenotypic traits in genetic breeding programs. GWAS was used to enhance genetic breeding in various animals, i.e., cattle, sheep, pigs and chickens [14,15]. GWAS has discovered genetic differences related to economically essential traits such as production qualities [16], milk production traits [14], body conformation [17], reproduction traits [18], and mastitis [19,20]. The genetic correlation between body conformation traits such as udder depth and teat traits was studied for indicators of longevity and lifetime in Dutch dairy cattle [17]. A genome-wide association study has been approved to detect the SNP that is related to quantitative trait loci of dairy cattle [1,21]. In post-GWAS analyses such as gene ontology (GO), term analyses have given researchers a better understanding of the molecular mechanisms underlying conformation traits and have allowed them to identify the most likely candidate genes [18,22]. In beef cattle, genetic estimations and selection are improving due to traits that affect efficiency as well as animal fitness and wellbeing. The Canadian Angus Association (CAA) recently established a genetic analysis in teat and udder score for enhancing genetic selection in Canadian Angus cattle [1]. Ten candidate genes for teat- and udder-associated traits in French dairy cattle were identified [22].

The objective of our study is to identify the significant SNPs associated with the anterior teat position (ATP), posterior teat position (PTP), and front teat length (FTL) confirmation traits in Chinese Holstein cattle by using the GWAS approach, and to find the SNP position of candidate genes and pathways that affect these teat traits.

## 2. Materials and Methods

### 2.1. Ethics Statement

The Ministry of Agriculture and the China Council on Animal Care provided guidelines for collecting hair follicle samples and measuring phenotype traits. It was also accepted by the School of Yangzhou University Animal Research Group Institutional Animal Care under the research group license number: SYXK (Su) IACUC 2012-0029. Anesthesia was not used for experimental animals during this study.

### 2.2. Animal Phenotype Measurement and DNA Sample Collection

One thousand Chinese Holstein cattle were selected from 4 animal farms in the area of Jiangsu Province, China. The studied Holstein cattle population was raised on four dairy farms, Sihong farm: 199, Xuyi farm: 214, Xuzhou farm: 224, and Huaxia farm: 363 animals. The primary data were measured within two months, from November to December 2019. The three mammary system teat traits were measured individually, while teat traits were recorded on a scale from 1 to 9. Three mammary system teat-related traits, anterior teat position (ATP), posterior teat position (PTP), and front teat length (FTL), of the 1000 cattle were measured according to the China National Standard (GB/T 35568-2017); three specialists completed the phenotype trait measurements for each cow. For genotyping analysis, hair follicle samples were collected from each cow and were stored in special paper envelopes to prevent environmental and DNA contamination; thus, a minimum of 50 hairs were present in each paper envelope.

### 2.3. Phenotype and Genotype Parameters for Analysis

Pairwise Pearson coefficients and descriptive statistics of the phenotypic datasets were analyzed by using computer-based software, SPSS software (v16.0, Chicago, IL, USA). Genetic analysis was performed by using DMU software (v5.6, Aarhus, Denmark) to estimate individuals’ heritability and genetic correlation between pairs of traits in Holstein cattle [23]. The phenotype of density distribution of three traits was obtained by using R language (v 4.0.2, Auckland, New Zealand).

The three teat trait scores were used for the following analysis, and the following two steps were used for all calculations, adjusted with fixed environmental factors. The pedigree data were measured back to three generations (2010–2020); the parities of the cattle were between 1 and 4.

**Steps 1**. Multiple-trait animal models were used to estimate genetic parameters:

(1) y=Xb+Za+e

where y shows the phenotype of an individual; X is the design matrix for fixed effects (farm, age and parity); b shows the vector of fixed effects; Z is a matrix to link a with y; a is the individual additive genetic effect; and e shows the vector random residuals. The variance components of $\sigma_{a}^{2}$ and $\sigma_{e}^{2}$ were assessed by means of the REML method in DMU software, according to the method presented by Madsen et al. [23]. The heritability of each trait was estimated as $h^{2}=$ $\sigma_{a}^{2}/\left(\sigma_{a}^{2}+\sigma_{e}^{2}\right)$.

**Steps 2**: We used the following model for adjusted phenotype:

(2) $$y_{adj}=y-X\hat{b}$$

where $y_{adj}$ shows the vector of adjusted phenotype traits; y, and X are the same as in Equation (1), and $\hat{b}$ is an estimate of b, which was estimated in Equation (1).

### 2.4. Genotyping and Quality Control

The DNA of the hair follicles was extracted and genotyped by using the GGP (Geneseek Genomic profiler) Bovine 100K SNP Chip by Neogen Biotechnology (Shanghai, China, http://www.neogenchina.com.cn, accessed on 25 July 2021), and the ARS-UCD1.2 (bosTau9) was used as a genome reference. The GGP Bovine Chip contained approximately 100,000 high polymorphic SNPs that were used for individual genotyping data. Meanwhile, quality control (QC) was conducted by using Plink software (v1.90, Cambridge, MA, USA) to remove the markers that did not meet the following standards [24]: (1) the call rate of an individual genotype was lower than 95%, (2) the call rate of single SNP genotype was less than 90%, and (3) the minor allele frequency (MAF) of SNP was >0.05 and deviated from the Hardy–Weinberg equilibrium value (*p* < 1.0 × 10^−6^). After quality control, there remained 984 cows and 84,406 SNP variants for further association analysis.

### 2.5. Population Structure Analysis

We used Plink software (v.1.90, Cambridge, MA, USA) to detect principal component analysis (PCA) on 984 cows genotyping [24], with 84,406 SNPs of Holstein cattle raised at four dairy farms, to investigate the population structure. Principal component analysis was plotted using R language (v 4.0.2, Auckland, New Zealand).

### 2.6. Genome-Wide Association Analysis

We conducted GWAS analysis; the fixed and random model circulating probability unification (FarmCPU) model used a multi-locus linear mixed model to carry out the association analysis between SNPs and traits [25]. In a fixed effect model, the FarmCPU method conducts the marker tests with associated markers as covariates and optimization on the associated covariate markers in a random effect model [25]. As we identified in association studies, population stratification is a significant factor that can lead to false positives [26]. Therefore, in this study, the first three PCs were used as covariate variables in the GWAS models [25], and the fixed effect model formula is given.

(3) $$y=Xb_{X}+M_{t}b_{t}+S_{j}d_{j}+e$$

where y is an adjusted phenotype of traits; $b_{t}$ is the fixed effect of the t pseudo QTN and $M_{t}$ is the corresponding genotype matrix; $S_{j}$ shows the genotype of the marker j; $d_{j}$ shows effect of the marker j; and e is a vector of random residuals with a distribution with zero mean and variance of $\sigma_{e}^{2}$. After substitution, every marker has its own *p* value. The associated marker map and *p* values are utilized to update the selection of pseudo-QTNs by using the SUPER algorithm in a REM, as follows [27]:

(4) y=u+e

where y and e show the same fixed effect model, and u satisfies the formula $u\;\sim \;N\;\left(0,K\sigma_{u}^{2}\right)$, in which u shows genetic effects in an individual. We identified the significance of threshold value for selecting the significant SNPs by using the method of Bonferroni correction [28]. The genome-wide significant threshold value was 5.92 × 10^−7^ (0.05/84,406).

### 2.7. Candidate Gene Identification

We determined the candidate gene and genomic region by using the UCSC genome browser from cow assembly April 2018 (http://genome.ucsc.edu, ARC-UCD1.2/bos Tau9, accessed on 25 July 2021) [29] and the National Center for Biotechnology Information gene “NCBI” (http://www.ncbi.nim.nih.gov/gene/) database (accessed on 25 July 2021).

### 2.8. Gene Pathway Enrichment Analysis

In this study, we submitted the candidate genes attained by GWAS into the profiler. The gene ontology (GO) [30] and the Kyoto Encyclopedia of Genes and Genome (KEGG) pathway analyses [31] (https://www.genome.jp/kegg, accessed on 25 July 2021 ) were conducted on the candidate gene by using the cluster profiler package in R software(v 4.0.2, Auckland, New Zealand) [32].

## 3. Results

### 3.1. Description and Heritability of Traits

**Figure 1:**
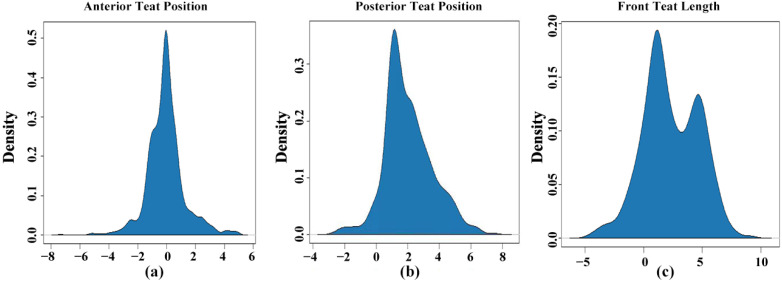
Density plot; division adjusted of phenotypes ATP (**a**), PTP (**b**) and FTL (**c**) in the population of Holstein cattle.

Phenotypic data of 1000 animals were used for description data analysis. The mean and standard error (S.E) of ATP were 5.34 (0.33), those of PTP were 6.50 (0.51) and those of FTL were 5.72 (0.52), while the estimated range of the mean (SD) and standard error was1.04 to 1.63 for teat traits. The distribution of teat trait score is shown in Figure S1, while the density plot shows the adjusted phenotype distribution among the teat traits (Figure 1). Heritability was estimated for the mammary system-related teat traits ATP, PTP and FTL, at 0.17, 0.37, and 0.13, respectively, among the different types of traits (Table 1).

### 3.2. Phenotype and Genotype Correlation

Similarly, the directions of pairwise phenotypic correlations were estimated by the Pearson correlation coefficient in mammary system-related teat traits. The estimates of positive phenotypic correlations were 0.16 (FTL and ATP), 0.27 (PTP and ATP), and 0.03 (FTL and PTP) for teat traits (Table 2). The pairwise genetic correlation was estimated between pair traits by DMU software (v5.6, Aarhus, Denmark). The pairwise genetic correlations were 0.48 (FTL and ATP), −0.09 (PTP and ATP), and 0.24 (PTP and FTL) for teat traits, while PTP and ATP showed a negative correlation between traits (Table 2).

### 3.3. Information of Marker (SNPs)

After quality control, 984 cattle and 84,406 SNPs were used to perform GWAS, and the filter SNPs were distributed on 29 chromosomes. After quality control, the minor allele frequency of all SNPs was recalculated due to its low value, and only MAF remained above 5% in the data. The distribution of the SNP information on chromosomes is shown in Figure S2.

### 3.4. Population Structure Analysis

**Figure 2:**
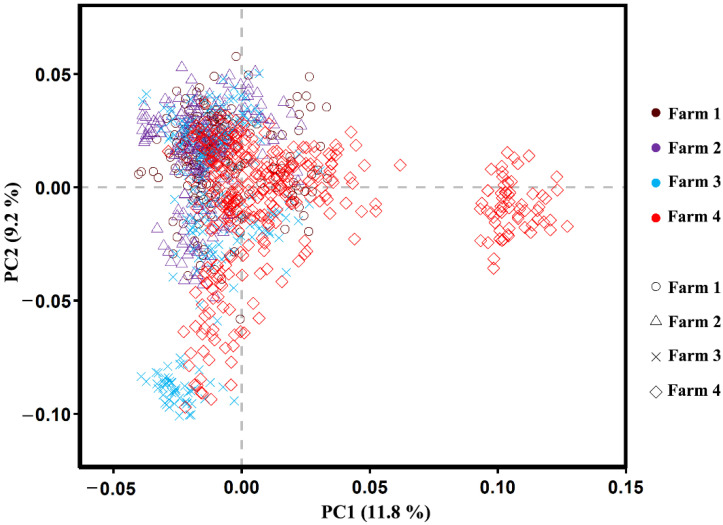
Population structure plots revealed by the 84,406 SNPs of the 984 cattle. Principal component analysis (PCA). PC1 and PC2 contributed 11.8% and 9.2% −of the variation, respectively.

The PC analysis was used to formulate the population structure of the 984 animals in four farms: farm 1: 197, farm 2: 206, farm 3: 220, and farm 4: 361 animals. The population structure (Figure 2) showed all results in the four groups as a cluster. PCA analysis was used to detect the level of population stratification. Therefore, these principal components were performed as covariates in the fixed effect model for association analysis to prevent false positives caused by group stratification. Principal components explained 21% of the variation, in which PCA1 and PCA2 contributed 11.8% and 9.2% of the genomic relationships, respectively; therefore, the first three PCs in the principal component analysis were fixed as variable covariates in the association analysis using the FarmCPU model.

### 3.5. Results of Genome-Wide Associations

**Figure 3:**
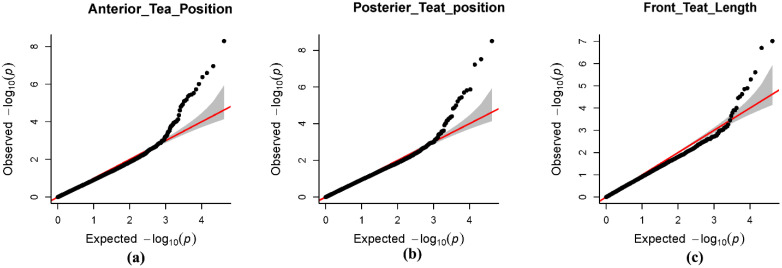
Quantile–quantile plot of the three traits. (**a**), anterior teat position (ATP); (**b**), posterior teat position (PTP); and (**c**), front teat length (FTL) from GWAS in Holstein cattle. Q–Q plot showed the observed and expected *p* value of every SNP. The red line shows no association of null hypothesis. The black dot SNP showed the threshold.

**Figure 4:**
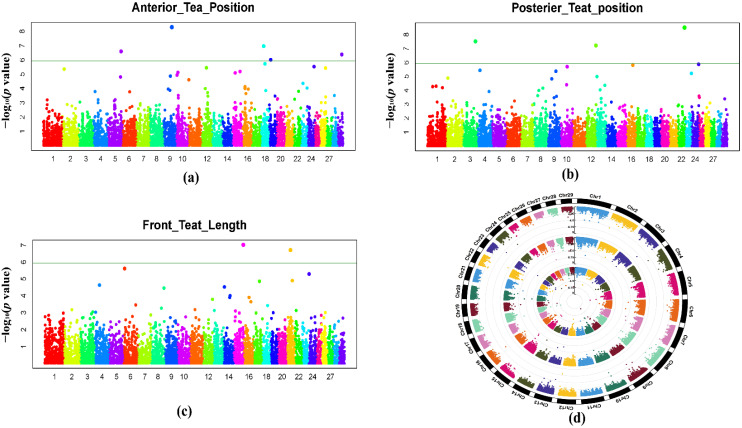
Manhattan plots of (**a**) ATP, (**b**) PTP and (**c**) FTL traits showed the detected *p* value of each SNP from GWAS in Chinese Holstein cattle. Manhattan plots in which the genomic coordinate of SNPs is shown along the horizontal axis. On the vertical axis, the negative logarithms of the association of *p* value of each SNP are represented, and after the Bonferroni correlation, the green horizontal lines indicate significant threshold value. Circular Manhattan plot: the three teat shape conformation traits were plotted from outside to inside (**d**).

GWAS analysis was conducted by using the FarmCPU model in this study. The quantile–quantile plots (Figure 3) showed that the model used in this research for GWAS analysis was suitable. The lambda values (λ) of ATP, PTP, and FTL were 0.97, 0.95, and 0.92, respectively, and they were all close to 1.01, and the line certified the accuracy of the association analysis between traits and SNPs. In the Q–Q plots, the point in the upper right corner showed the significant SNPs that were associated with traits under research (Figure 3). The Manhattan plots (Figure 4) were used to show the results of GWAS significance level (−log10 of *p* value of each SNP) by chromosome position. Large peaks of SNPs in the Manhattan plot showed a strong association with traits.

In the GWAS study, the threshold value for significant SNP showed 5.92 × 10^−7^ (0.05/84406). All nine significant SNPs were associated with mammary system-related teat traits. Four significant SNPs were located on chromosomes Chr9 (ARS-BFGL-NGS-101241), Chr29 (ARS-BFGL-NGS-43147), Chr18 (BovineHD1800006781) and Chr5 (BovineHD0500031672), which were detected to be associated with trait ATP. For posterior teat position, three significant SNPs were detected on chromosomes Chr22 (ARS-BFGL-NGS-16048), Chr3 (Hapmap58721-rs29026738), and Chr12 (12-88054488-G-A-rs4235240); these SNPs were related to trait PTP. For front teat length (FTL), two SNPs were located on the chromosomes Chr15 (BovineHD1500023818) and Chr21 BovineHD2100009187, which were detected to be associated with trait FTL (Table 3).

### 3.6. Identification of Candidate Gene

In this study, of the genes that are located at a distance within this region, 200 kb of significant SNPs are known as candidate genes. All nine significant SNPs were associated with traits. The five SNPs, BovineHD0500031672, ARS-BFGL-NGS-16048, Hapmap58721-rs29026738, 12-88054488-G-A-rs42352402, and BovineHD1500023818, were located within the genes peroxisome biogenesis factor 26 (*PEX26*), mitochondrial trans-locator assembly and maintenance protein 41 homolog (*TAMM41*), HIVEP zinc finger 3 (*HIVEP3*)*,* myosin 16 (*MYO16*), and SET binding factor 2 (*SBF2*), respectively (Table 3). The four single-nucleotide polymorphisms ARS-BFGL-NGS-101241, ARS-BFGL-NGS-43147, BovineHD1800006781 and BovineHD2100009187 were located within 100 kb distance of E2F transcription factor 8 (*E2F8*), cysteine and glycine rich protein 3 (*CSRP3*), cadherin 11 (*CDH11*) and syntaxin binding protein 6 (*STXBP6*), respectively, whereas the four SNPs were found on different chromosomes.

### 3.7. Bioinformatics Analysis

UCSC Genome browser and the NCBI database were used for identifying genes through cow assembly in April 2018 (ARC-UCD1.2/bosTau9). A total of 185 genes were found within a 200 kb region on significant SNPs for the mammary system-related teat traits, and these functional genes were used for further analysis, gene ontology terms and KEGG pathway analysis.

### 3.8. Gene Enrichment Analysis

**Figure 5:**
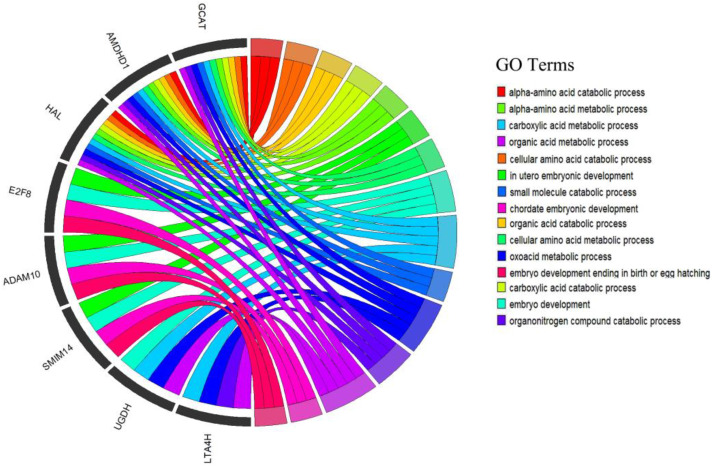
Gene ontology term results of candidate gene in mammary system-related teat shape confirmation traits.

For a deeper understanding of the functions of the nine significant SNPs that are related to indicators of the mammary system performance of cattle, the genes within 200 kb of significant SNPs for each mammary system-related teat shape trait were selected for functional enrichment analysis. A total of 185 genes were used for gene enrichment analysis. GO and KEGG analysis were conducted by the Cluster Profiler Software Package in R (v 4.0.2, Auckland, New Zealand). Gene ontology results were significantly enriched (*p* < 0.05) in teat traits, and gene ontology enrichment analysis derived 36 GO terms, which included 28 biological processes, one cellular component and seven molecular functions, as shown in Supplementary Table S1. The GO result shows candidate genes that are related in GO terms (Figure 5).

KEGG results (Table 4) identify that each teat trait of the candidate genes was enriched in the following four pathways: allograft rejection (bta05330), apoptosis (bta04210), cytokine–cytokine receptor interaction (bta04060), and histidine metabolism (bta00340); thus, 20 of the 185 candidate genes were involved in the pathway of regulation. KEGG pathways were selected based on the *p* value (*p* < 0.05), in which all pathways are closely related to mammary system-related teat traits. A KEGG pathway dot plot is shown in the Supplementary Materials (Figure S3).

## 4. Discussion

Mammary system-related teat traits are a type of linear trait or functional trait in dairy cattle. These traits are associated with cattle longevity, health and welfare. In this study, the heritability of mammary system-related teat traits of Holstein cattle was of a medium level (Table 1). Previous studies have identified that animal differences in bovine mammary systems are genetically influenced by the heritability value (estimate range between low to medium) [1,33]. Wu et al., 2013, estimated the heritability of front teat placement, teat length and rear teat placement (0.18, 0.21 and 0.11, respectively) in Chinese Holstein cattle [34]. The heritability of the three mammary system-related teat traits studied in this research was between 0.13 and 0.37 (ATP 0.17, PTP 0.37 and FTL 0.13; Table 1) in Chinese Holstein cattle.

The relationship between phenotypic and genotypic correlations has been researched in animal studies [35]. Front teat placement (FTP) showed genetic correlations (−0.33) for Holstein breeds [22]. A notable exemption was the individual fore udder (FU) trait, recording a negative to moderately positive genetic correlation that ranged from −0.39 to 0.35 between fore teat placement (FTP) and fore teat length (FTL) in Chinese Holstein cattle [36]. Phenotype and genetic correlation were estimated in the mammary system-related teat traits of cattle. Estimates of positive phenotypic correlations of FTL and ATP, PTP and ATP, and PAT and FTL were 0.16, 0.27, and 0.03, respectively, for mammary system-related teat traits. Estimates of genetic correlations were measured between mammary system-related teat traits. Genetic correlations of FTL and ATP, PTP and ATP, and PTP and FTL were 0.48, −0.09, and 0.24, respectively, for teat traits, while posterior teat position and anterior teat position showed a negative genetic correlation between traits (Table 2).

Population stratification is a major challenge faced in GWAS studies. Population stratification and family structure can cause several false positive results in GWAS [37]. In GWAS studies, population stratification is a critical causative factor. When various genetic structures are involved, genetic variations caused by the selection of cows from various groups and areas may be interpreted as phenotypic differences in the GWAS practice, leading to false positive associations. [38]. After proper correction, the lambda (λ) value should be close to 1 [39]. There are numerous methods for accurate stratification of the population, and the statistical model can be a useful method to correct and minimize the chances of type-1 errors (false positive associations) [40]. The general linear model was conducted using a suitable population structure as a covariate [41], and the mixed linear model used both a population structure by filtering and the total genetic effect in each individual as covariates. Effective ways to control or minimize false positives and make adjustments for testing markers have been suggested. Mixed linear model (MLM) can miss potentially important discoveries and lead to false negatives due to the confounding between population structures, quantitative trait nucleotides (QTNs), and kinship [42]. Therefore, in this research, we performed the FarmCPU model analysis for totally false positive controls, removed confounding, and improved the computational power of efficiency by using the fixed effect model and random effect model, iteratively [25]. In population-based association studies, population stratification is a critical issue [43,44].

After the correction of population stratification, the inflation factor mean lambda value should be close to 1 [42]. According to previous studies, the lambda value (λ) was 0.983 and 1.004 for colostrum and serum albumin concentrations, respectively, indicating that population stratification was successfully corrected by the appropriate model [45]. In the present study, the Q–Q plots of teat traits (Figure 4) showed that the deviation of the observed value from the expected value is near 1 and that the inflation factor, or lambda value (λ), of ATP, PTP and FTL traits was close to 1.01. These factors indicate that population stratification was corrected by using different suitable models.

The principal component analysis method [44] detected the population structure that divides individuals into grouped ancestry based on their genetic character (Figure 2), and the experimental population was clustered into four groups, where different colors of the clusters showed each animal’s farm. Each group that was closely clustered had a stronger genetic relationship in the animal population. This division of four groups was indicated in the semen and blood, and these four groups may be attributed to Holstein semen from different overseas countries. These cattle contained blood from other breeds; therefore, we know that Chinese Holstein cattle can be registered (Chinese Holstein, GB/T 3157 2008) as at least having 87.5% foreign blood in the Holstein population. The stratification could be because the semen used on the four animal farms came from multiple countries, and this semen came from native bulls. In all animals, the genetic population structure was involved as a fixed effect in the model GWAS to correct the effects of population stratification. Therefore, the PCA plot showed a population structure and also showed variation in genomic relationships of population structure.

In the present study, GWAS analysis found nine significant SNPs that are associated with three traits (ATP, PTP and FTL) in Chinese Holstein cows by using the FarmCPU model. The two most highly significant SNPs (ARS-BFGL-NGS-101241 and ARS-BFGL-NGS-16048) occurred among them; two SNPs were located nearby: the *MMS22L* gene within *TAMM41.* These genes were associated with ATP and PTP traits. *MMS22L* may help in homologous recombination-mediated genome stability maintenance during DNA replication [46]. River buffalo are primarily selected for milk production and are well adapted to hot climates; *MMS22L* genes might play an essential role in river buffalo’s heat adaptability [43]. Nguyen et al. [47] reported that the *MMS22L* gene has been detected in esophageal cancers and clinical lungs of humans. Kutik et al. [48] described that the *TAMM41* gene is the mammalian equivalent of a yeast protein; the deletion of the Tamm41 gene resulted from the high content of phosphatic acid in mitochondria. The first metabolite was involved in the biosynthesis of cardiolipin, with a virtual absence of phosphatidylglycerol in most cardiolipin species.

The study recognized the other genes that are significantly associated with anterior teat position, posterior teat position and front teat length traits (*PEX26*, *MYO16*, *HIVEP3* and *SBF2*). These candidate genes of teat traits have effects on mammary structure and milk production in dairy animals. Expression of *PEX26* gene mutations have occurred in Chinese hamster ovary cells and human cell lines and can cause disorders of peroxisome biogenesis (PBDs) in humans [49]. The *MY016* gene affects milk production yield, fat yield and protein yield in Buffalo animals [50]. The *MY016* gene (BTA12) has been identified at significant SNPs within position in Holstein cattle [51]. The finding of this mutation in the *SBF2* gene may refer to the cause of thrombocytopenia in humans [52]. The *HIVEP3* gene belongs to the enhancer-binding protein family of the human immunodeficiency virus type 1 that encodes large zinc finger proteins and regulates transcription via the kappa B enhancer motif [53]. In prostate cancer, the *HIVEP3* gene functions as an oncogene [54].

The *CDH11* and *STXBP6* genes were identified and are associated with ATP and FTL in Holstein cattle population. The significant SNP (BovineHD1800006781) was located on Chr18 and had a position within 100 kb of the cadherin (*CDH11*) gene. Specifically, the highly associated *CDH11* gene belonging to this module has been identified in the development of mastitis and growth of mammary glands in dairy animals [55,56]. *STXBP6* has important roles in the development of multiple diseases, including neurological disorders, diabetes, and cancer in humans and animals [57]. The significant candidate gene of *E2F8* is important for orchestrating genes essential for cell cycle progression and proliferation [58]. The present study exposed new hormonal and developing regulations of E2F8 mRNA during bovine follicular growth, while the *E2F8* gene has been found in the follicular development of cattle [59]. In cattle, the *CSRP3* gene is found on chromosome 29, and these findings suggest *CSRP3*’s high potential as a marker gene for growth improvement and body traits in the selection program of Chinese Holstein cattle [60]. *CSRP3* genes have been functionally characterized and play vital roles in the development of tissue-specific cell growth in sheep [61].

In our study, gene ontology terms enrichment analysis and KEGG pathway analysis identified a number of GO terms and KEGG pathways. The identified significant genes were related to our traits under observation; for example, the four candidate genes (*MMS22L*, *E2F8*, *HAL* and *STXBP6*) were clustered in biological processes, and these genes were associated with our significant SNPs. *MMS22L* gene terms were related to DNA-dependent DNA replication (GO: 0006261) and recombination DNA repairing (GO: 0000725); *E2F8* gene terms were related to embryo development (GO: 0009790) and utero embryonic development (GO: 0001701); *HAL* gene terms were related to cellular amino acid catabolic processes (GO: 0009063), cellular amino acid metabolic processes (GO: 0006520), and organic acid metabolic processes (GO: 0006082); and *STXBP6* gene terms were related to proteins containing complex subunit organization in biological processes. This is important for cell division during the growth and repair of damaged tissues; in mammals, the disruption of DNA replication can lead to a loss of genome integrity and an increase in cancer susceptibility [62]. The differential metabolites involved in pathways, including amino acids, nucleotide metabolism, carbohydrate, energy, lipid, cofactors, and vitamins and biosynthesis of other secondary metabolites are associated with the development of mammary glands, lactogenesis, and lactation in yaks, which were described by Li et al. [63]. The *HAL* gene was found in gene ontology and has an effect on amino acid metabolism in animals and humans; dietary histidine is related to factors that improve metabolic syndrome and affect ion absorption in human populations. In ruminant animals, histidine is a limiting factor for milk protein synthesis, and it may be the first limiting amino acid for growth [64]. In Chinese Holstein cattle, the *HAL* gene was found to be significantly associated with milk protein yield and milk yield. Additionally, the *HAL* gene has been reported within QTLs for milk production traits [27].

The *UGDH* gene works as coenzyme binding (GO: 0050662). The UDP-glucose 6-dehydrogenase (*UGDH*) gene had been confirmed to be related to biological processes, metabolic processes, growth performance, and milk production in Holstein cattle [65].

The top four significant KEGG analysis pathways were related to immune response, e.g., allograft rejection (KEGG: bta5330), which has three candidate genes; apoptosis (KEGG: bta04210), which consists of seven genes; cytokine–cytokine receptor interaction (KEGG: bta04060), which comprises eight genes; and histidine metabolism (KEGG: bta00340), which is a non-significant pathway that has two genes. All candidate genes are involved in the regulation of pathways and mammary development; one candidate gene, *FASLG*, is commonly found in the first three KEGG pathways. The *FASLG* gene has a role in apoptosis in normal human mammary cells [66]. The expression of immune response-related *BoLA–DRA* genes influences the immune function of lactating cows and decreases milk yields [67]. While the *HAL* gene was found in both gene ontology terms and KEGG pathways, histidine metabolism is a limiting factor for milk protein synthesis, and it may be the first limiting amino acid for growth in animals (cattle, poultry, fish and humans) [64]. Therefore, it is practical to suppose that all significant SNPs and candidate genes might be associated with mammary system-related teat traits.

## 5. Conclusions

GWAS analysis was performed on three mammary system-related teat traits, which were chosen as indicators of mammary system performance in Chinese Holstein cattle. This study identified nine significant SNPs associated with ATP, PTP and FTL traits in Chinese Holstein cattle. We also found several candidate genes, *MMS22L*, *E2F8*, *CSRP3*, *PEX26*, *CDH11*, *HAL*, *TAMM41*, *HIVEP3*, *MYO16*, *SBF2* and *STXBP6*, that contribute to metabolism, immune response, biological information, and genetic improvement. Our study highlighted useful biological information to understand genetic architecture and provided fundamentals for molecular breeding programs that can lead to genetic improvement programs regarding ATP, PTP and FTL traits in Holstein cattle. Further research is needed to confirm the biological activities of these genes.

## Figures and Tables

**Table 1:** Description of data analysis and heritability of cattle mammary system-related teat traits.

Traits	Mean	Std. Error	Std. Deviation	Minimum	Maximum	Heritability
ATP	5.34	0.33	1.04	1	9	0.17
PTP	6.50	0.51	1.62	1	9	0.37
FTL	5.72	0.52	1.63	2	9	0.13

Mean, std. Error and SD; standard deviation score of teat traits and heritability (grey line) for anterior teat position (ATP), posterior teat position (PTP), and front teat length (FTP) trait measurements.

**Table 2:** Pearson phenotypic (upper diagonal) and genetic (lower diagonal) correlations for ATP, PTP and FTL traits in Chinese Holstein cattle.

Traits	ATP	PTP	FTL
ATP	1	0.27 **	0.16 **
PTP	−0.09	1	0.03
FTL	0.48	0.24	1

ATP, Anterior teat position; PTP, posterior teat position; FTL, front teat length. The superscript ** shows a significant correlation at 0.01.

**Table 3:** Genome-wide significant SNPs associated with mammary system-related teat confirmation traits.

Traits	SNP Name	CHR	Position (bp)	MAF	Nearest Gene	Distance (kb)	*p* Value	Effect
ATP	ARS-BFGL-NGS-101241	9	67971873	0.24	*MMS22L*	100kb	5.10 × 10^−9^	−0.22498
	ARS-BFGL-NGS-43147	29	37332830	0.12	*E2F8*	100kb	4.16 × 10^−7^	−0.24097
	BovineHD1800006781	18	22013861	0.48	*CDH11*	100kb	1.09 × 10^−7^	0.16219
	BovineHD0500031672	5	109356610	0.18	*PEX26*	Within	2.54 × 10^−7^	−0.20494
PTP	ARS-BFGL-NGS-16048	22	55808310	0.34	*TAMM41*	Within	3.14 × 10^−9^	−0.50077
	Hapmap58721-rs29026738	3	104278203	0.084	*HIVEP3*	Within	3.05 × 10^−8^	−0.83939
	12-88054488-G-A-rs42352402	12	84040288	0.168029	*MYO16*	Within	6.02 × 10^−8^	0.61091
FTL	BovineHD1500023818	15	80481458	0.37	*SBF2*	Within	9.69 × 10^−8^	0.27188
	BovineHD2100009187	21	31317530	0.46	*STXBP6*	100kb	1.98 × 10^−7^	0.28855

SNP, single-nucleotide polymorphism; CHR, chromosome; ATP, anterior teat position; PTP, posterior teat position; FTL, front teat length; MAF, minor allele frequency; Effect.

**Table 4:** KEGG pathway analysis using candidate genes associated with genome-wide significant association.

KEGG ID	Description	Gene Count	*p* Value	Genes Name
bta05330	Allograft rejection	3	3.68 × 10^−6^	*FASLG, BOLA-DRA* *GZMB*
bta04210	Apoptosis	7	4.50 × 10^−4^	*BID, FASLG, LOC505326, LOC786826, LOC508858, LOC508646, GZMB*
bta04060	Cytokine–cytokine receptor interaction	8	1.09 × 10^−2^	*CCL16, LOC616364, LOC100297044, LOC525415, TNFSF18, FASLG, TNFSF4, TNFSF8*
bta00340	Histidine metabolism	2	4.91 × 10^−2^	*AMDHD1, HAL*

## Data Availability

The data presented here are available on request.

## Supplementary Materials

The following are available online at https://www.mdpi.com/article/10.3390/genes12122020/s1, Distribution of Teat traits score, Figure S1; Distribution of SNPs, Figure S2; KEGG dotplot, Figure S3; GO Results, Table S1.
