# Supplementary material for: Genome-Wide Association Study Candidate Genes on Mammary System-Related Teat-Shape Conformation Traits in Chinese Holstein Cattle
Source: Genes (Basel). 2021 Dec 19;12(12):2020. doi: 10.3390/genes12122020 (PMC8701322; doi:10.3390/genes12122020)
Supplement: Supplementary file 1 [file genes-12-02020-s001.zip › genes-1431419-suppl.pdf]

Supplementary Files

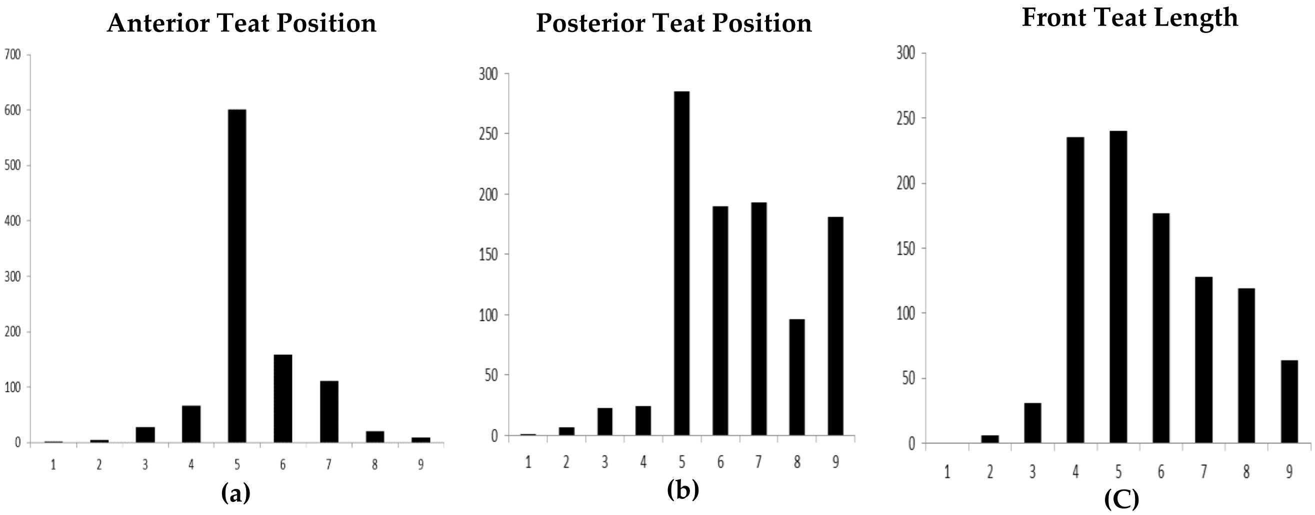

Figure S1. Distribution for Teat traits Condition Score (1-9) of the phenotypes of ATP (a), PTP (b) and FTL (c) of the Holstein Cattle.

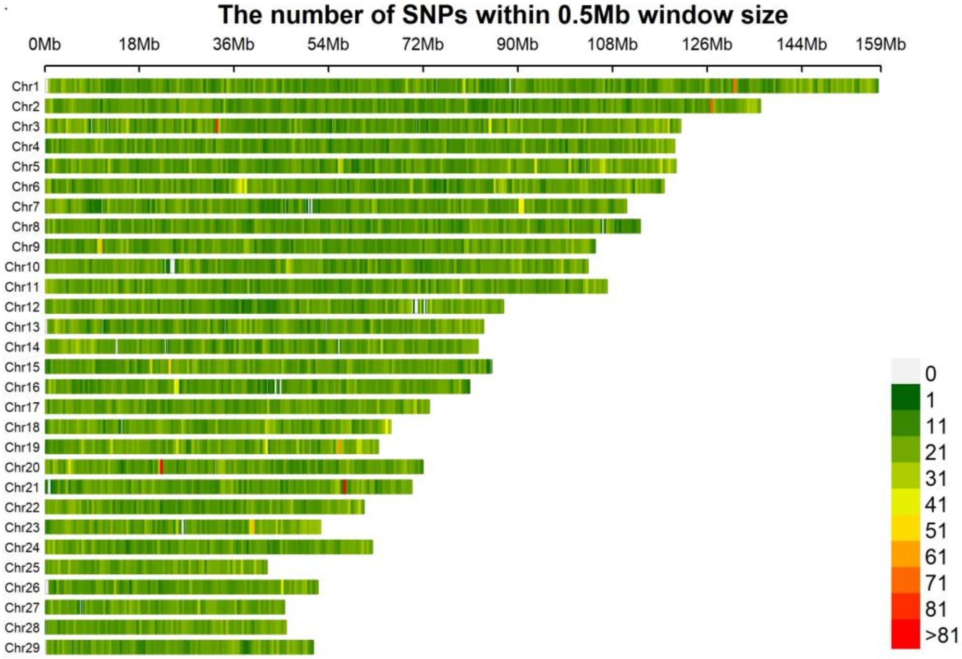

Figure S2. Distribution of SNPs is showed on 29 autosomes chromosomes in heatmap by MAF.

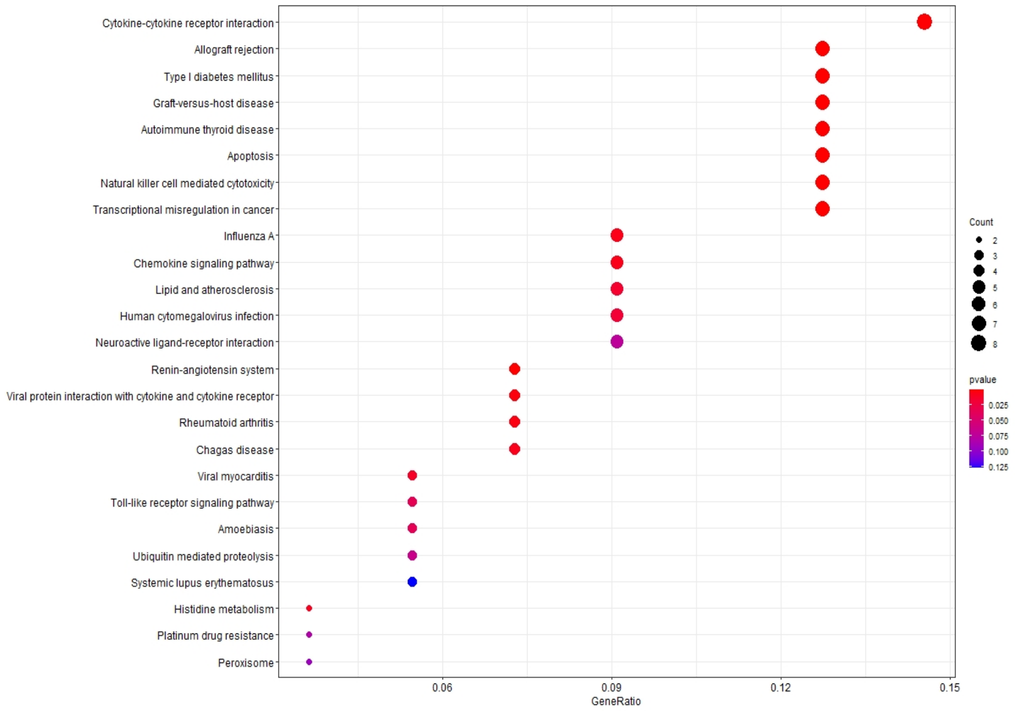

Figure S3. Kyoto encyclopedia of genes and genomes (KEGG) pathways analysis for the regional candidate gene with genome-wide association.

Table S1. Gene Ontology (GO) results

Gene Ontology	ID	Description	*p* value	Gene Name	Counts
Biological Process	GO:1901606	alpha-amino acid catabolic process	0.0013	*GCAT/AMDHD1/HAL*	3
	GO:0009063	cellular amino acid catabolic process	0.0017	*GCAT/AMDHD1/HAL*	3
	GO:0009072	aromatic amino acid family metabolic process	0.0022	*AMDHD1/HAL*	2
	GO:0006536	glutamate metabolic process	0.0026	*AMDHD1/HAL*	2
	GO:0070936	protein K48-linked ubiquitination	0.0068	*UBE2Q2/UBE2K*	2
	GO:0016054	organic acid catabolic process	0.0072	*GCAT/AMDHD1/HAL*	3
	GO:0046395	carboxylic acid catabolic process	0.0072	*GCAT/AMDHD1/HAL*	3
	GO:0043648	dicarboxylic acid metabolic process	0.0081	*AMDHD1/HAL*	2
	GO:1901605	alpha-amino acid metabolic process	0.0088	*GCAT/AMDHD1/HAL*	3
	GO:0000724	double-strand break repair via homologous recombination	0.0089	*MMS22L/RAD51D*	2

	GO:0001701	in utero embryonic development	0.0091	*E2F8*/*ADAM10*/*SMIM14*	3
	GO:0000725	recombinational repair	0.0096	*MMS22L*/*RAD51D*	2
	GO:0009064	glutamine family amino acid metabolic process	0.0128	*AMDHD1*/*HAL*	2
	GO:0016999	antibiotic metabolic process	0.0128	*AMDHD1*/*HAL*	2
	GO:0006261	DNA-dependent DNA replication	0.0145	*MMS22L*/*E2F8*	2
	GO:0006520	cellular amino acid metabolic process	0.0166	*GCAT*/*AMDHD1*/*HAL*	3
	GO:0009790	embryo development	0.0172	*E2F8*/*ADAM10*/*UGDH*/*SMIM14*	4
	GO:0019752	carboxylic acid metabolic process	0.0182	*GCAT*/*AMDHD1*/*HAL*/*LTA4H*/*UGDH*	5
	GO:0044282	small molecule catabolic process	0.0202	*GCAT*/*AMDHD1*/*HAL*	3
	GO:0043436	oxoacid metabolic process	0.0214	*GCAT*/*AMDHD1*/*HAL*/*LTA4H*/*UGDH*	5
	GO:1901565	organonitrogen compound catabolic process	0.0214	*GCAT*/*AMDHD1*/*HAL*/*LTA4H*/*UBE2K*	5
	GO:0006082	organic acid metabolic process	0.0233	*GCAT*/*AMDHD1*/*HAL*/*LTA4H*/*UGDH*	5
	GO:0043009	chordate embryonic development	0.0248	*E2F8*/*ADAM10*/*SMIM14*	3
	GO:0006302	double-strand break repair	0.0331	*MMS22L*/*RAD51D*	2
	GO:0000209	protein polyubiquitination	0.0344	*UBE2Q2*/*UBE2K*	2
	GO:0043933	protein-containing complex subunit organization	0.0384	*MICAL3*/*SNRPF*/*LIPC*/*STXBP6*/*UBE2K*/*UGDH*	6
	GO:0006310	DNA recombination	0.0384	*MMS22L*/*RAD51D*	2
	GO:0006260	DNA replication	0.0468	*MMS22L*/*E2F8*	2
Cell Component	GO:0005657	replication	0.0044	*MMS22L*/*RAD51D*	2
Molecular Function	GO:0070011	peptidase activity, acting on L-amino acid peptides	0.0096	*LTA4H*/*ADAM10*/*MINDY2*/*GZMB*	4
	GO:0008233	peptidase activity	0.0108	*LTA4H*/*ADAM10*/*MINDY2*/*GZMB*	4
	GO:0008237	metallopeptidase activity	0.0143	*LTA4H*/*ADAM10*	2
	GO:0050662	coenzyme binding	0.0272	*MICAL3*/*GCAT*/*UGDH*	3
	GO:0008201	heparin binding	0.029	*LOC504773*/*LIPC*	2
	GO:0140096	catalytic activity, acting on a protein	0.0386	*LTA4H*/*ADAM10*/*MINDY2*/*UBE2Q2*/*UBE2K*/*GZMB*	6
	GO:0005539	glycosaminoglycan binding	0.0419	*LOC504773*/*LIPC*	2
